# Functional comparison of exome capture-based methods for transcriptomic profiling of formalin-fixed paraffin-embedded tumors

**DOI:** 10.1038/s41525-021-00231-7

**Published:** 2021-08-12

**Authors:** Kyrillus S. Shohdy, Rohan Bareja, Michael Sigouros, David C. Wilkes, Princesca Dorsaint, Jyothi Manohar, Daniel Bockelman, Jenny Z. Xiang, Rob Kim, Kentaro Ohara, Kenneth Eng, Juan Miguel Mosquera, Olivier Elemento, Andrea Sboner, Alicia Alonso, Bishoy M. Faltas

**Affiliations:** 1 Department of Medicine, Division of Hematology and Medical Oncology, Weill Cornell Medicine, New York, NY USA; 2 Department of Clinical Oncology, Kasr Alainy School of Medicine, Cairo University, Cairo, Egypt; 3 Caryl and Israel Englander Institute for Precision Medicine, Weill Cornell Medicine, New York, NY USA; 4 Institute for Computational Biomedicine, Weill Cornell Medicine, New York, NY USA; 5 Genomic Resources Core Facility, Weill Cornell Medicine, New York, NY USA; 6 Department of Pathology and Laboratory Medicine, Weill Cornell Medicine, New York, NY USA; 7 Sandra and Edward Meyer Cancer Center, Weill Cornell Medicine, New York, NY USA; 8 Department of Cell and Developmental Biology, Weill Cornell Medicine, New York, NY USA

**Keywords:** Molecular medicine, Cancer genomics

## Abstract

The availability of fresh frozen (FF) tissue is a barrier for implementing RNA sequencing (RNA-seq) in the clinic. The majority of clinical samples are stored as formalin-fixed, paraffin-embedded (FFPE) tissues. Exome capture platforms have been developed for RNA-seq from FFPE samples. However, these methods have not been systematically compared. We performed transcriptomic analysis of 32 FFPE tumor samples from 11 patients using three exome capture-based methods: Agilent SureSelect V6, TWIST NGS Exome, and IDT XGen Exome Research Panel. We compared these methods to the TruSeq RNA-seq of fresh frozen (FF-TruSeq) tumor samples from the same patients. We assessed the recovery of clinically relevant biological features. The Spearman’s correlation coefficients between the global expression profiles of the three capture-based methods from FFPE and matched FF-TruSeq were high (rho = 0.72–0.9, *p* < 0.05). A significant correlation between the expression of key immune genes between individual capture-based methods and FF-TruSeq (rho = 0.76-0.88, *p* < 0.05) was observed. All exome capture-based methods reliably detected outlier expression of actionable gene transcripts, including *ERBB2, MET, NTRK1*, and *PPARG*. In urothelial cancer samples, the Agilent assay was associated with the highest molecular subtype concordance with FF-TruSeq (Cohen’s *k* = 0.7, *p* < 0.01). The Agilent and IDT assays detected all the clinically relevant fusions that were initially identified in FF-TruSeq. All FFPE exome capture-based methods had comparable performance and concordance with FF-TruSeq. Our findings will enable the implementation of RNA-seq in the clinic to guide precision oncology approaches.

## Introduction

RNA sequencing (RNA-seq) has provided deep insights into gene expression patterns in biological samples, including transcript abundance levels, isoform expression, alternative splicing, and chimeric transcripts resulting from gene fusions. There is growing interest in leveraging RNA-seq as a clinical tool, especially in oncology, to match patients to targeted therapy and improve outcomes^1–5^. One of the barriers to the clinical implementation of RNA-seq is the need for fresh-frozen tumor samples to obtain optimal results. However, in the clinical setting, the vast majority of specimens are preserved as formalin-fixed, paraffin-embedded (FFPE) tissues for long-term storage. Unfortunately, this preservation process is associated with a rapid decline in RNA quality^2^. Several adverse factors impact the quality of RNA extracted from FFPE, including ischemia, formaldehyde fixation, embedding in warm paraffin, and the duration of the storage of tissue blocks^6,7^.

RNA capture is potentially more suited to the transcriptomic analysis of FFPE tumor samples^8^. Recently, several commercial RNA capture kits have become available. However, a systemic comparison of their ability to recover clinically relevant biological features from real-world FFPE samples has not been performed. The lack of an optimal method for transcriptomic profiling of FFPE tumor samples has hindered clinical application. To address this knowledge gap, we compared the sequencing metrics and biological readouts from the Agilent SureSelect V6 (Agilent), TWIST NGS Exome (TWIST), and IDT XGen Exome Research Panel (IDT) capture-based methods from FFPE tumor samples. For each sample, we compared the three FFPE capture-based methods to TruSeq RNA-seq of the fresh frozen (FF) sample from the same tumor (hereafter referred to as FF-TruSeq). We studied the potential clinical utility of FFPE capture-based methods to discover clinically useful readouts. The comparison focused on genes with outlier expression, the expression of key immune genes, molecular subtype classification, and the detection of gene fusions.

## Results

### Overview of the study

**Fig. 1:**
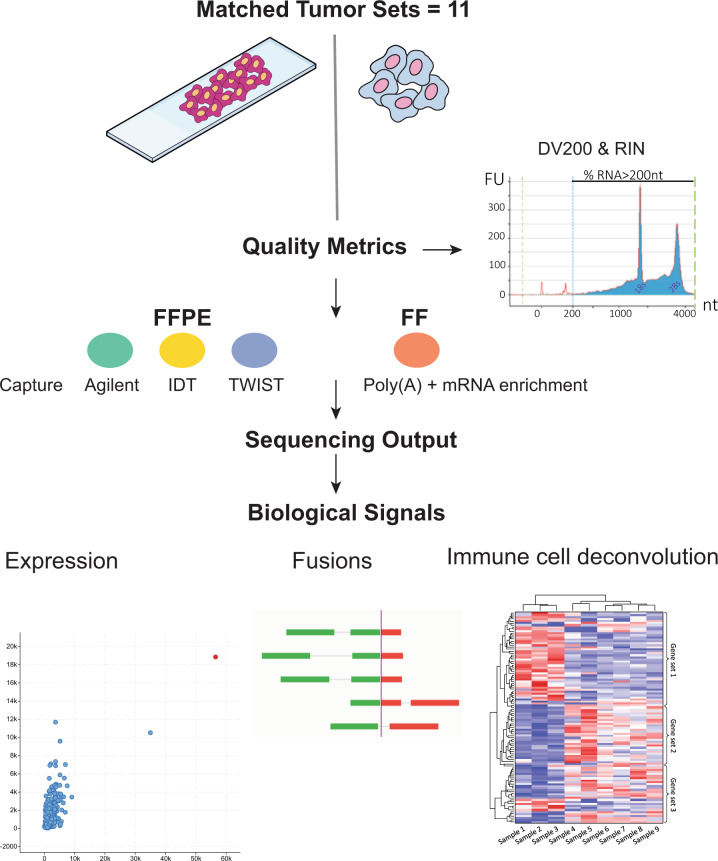
Study overview. Three exome capture-based methods (Agilent, TWIST, IDT) were used to construct sequencing libraries from FFPE tumor samples and were compared. The performance of the three capture-based methods in identifying readouts, such as outlier gene expression, fusions, and immune gene expression was benchmarked against FF-TruSeq of their matching fresh frozen samples from the same respective tumor. RIN RNA integrity number, nt nucleotides.

We designed this study to answer two main questions: First, what are the differences in the performance characteristics between the three commercially available FFPE capture-based methods (Agilent, TWIST, IDT)? Second, what are the performance characteristics of FFPE capture-based methods compared to TruSeq RNA-seq of matched FF tumor samples (Fig. 1)?

To answer these questions, we compiled a cohort of 32 FFPE tumor samples from 11 patients. For each patient, a matching FF tumor sample was available. We included several tumor types, namely urothelial cancer, gastroesophageal junction (GEJ) adenocarcinoma, oligodendroglioma, cancer of unknown primary (CUP), leiomyosarcoma, papillary thyroid cancer, and colorectal cancer (Supplementary Data 1). We performed RNA-seq (capture-based methods and TruSeq) of FFPE and FF tissues from the same tumor samples (Fig. 1).

### Alignment statistics

**Fig. 2:**
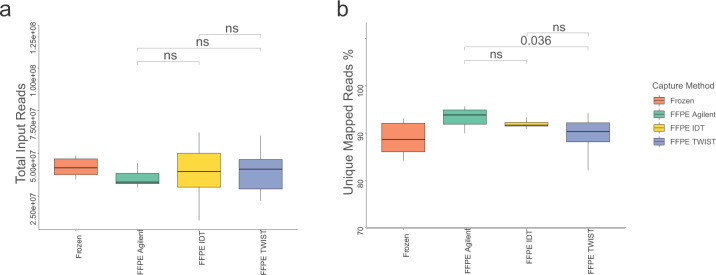
Sequencing outputs were comparable among the three capture-based methods. **a** Boxplots comparing the number of total input reads show no significant difference among the three exome capture-based methods. **b** Boxplots comparing uniquely mapped reads percentages among the capture-based methods. The centerline of each box is the median; the lower and upper bounds of the box are the first quartile (Q1) and the third quartile (Q3): the lower and upper whiskers show the minimum and maximum values not exceeding 1.5x interquartile range (IQR).

To compare the performance of FFPE capture-based methods, we analyzed the mapping statistics and compared them with those obtained by TruSeq of the matching FF tumor samples. The mean number of input reads was 38.6 million for FFPE capture-based methods and 44.4 million for FF-TruSeq. The mean number of input reads was not significantly different between the capture-based methods (Fig. 2a and Supplementary Data 2). The mean total number of uniquely mapped reads was 35 million for FFPE capture-based methods and 39 million for FF-TruSeq. The mapped reads percentage (the ratio of mapped reads to the input reads) was high for FFPE capture-based methods (mean 91.33%, SD = 3.20) (see “Methods” section). Across the FFPE capture-based methods, the mapped reads percentages were comparable between Agilent and IDT (Wilcoxon rank *p* > 0.05) and IDT and TWIST (Wilcoxon rank *p* > 0.05) (Fig. 2b). TWIST was associated with a significantly lower percentage of mapped reads (89%) compared to Agilent (94%) (Wilcoxon rank *p* = 0.03) (Fig. 2b). The percentage of multi-mapped reads was low across all FFPE capture-based methods (mean 3.44%, SD = 1.71). The Agilent capture method was associated with the lowest percentage of multi-mapped reads (2%) compared to IDT (5%, Wilcoxon rank *p* = 0.0001) and TWIST (3%, *p* < 0.0001) (Supplementary Fig. 1a and Supplementary Data 2). Collectively, the mapping metrics were comparable across the capture-based methods and FF-TruSeq.

### Global mRNA expression

We measured the mRNA expression levels using FPKMs (fragments per kilobase of exon model per million reads mapped) from the capture-based methods. TWIST showed the highest median log FPKMs compared to IDT (*p* < 0.0001) and Agilent (*p* < 0.0001) (Supplementary Fig. 1b). We examined the distribution of FPKMs from the capture-based methods. The mRNA gene expression from FF RNA-seq is known to follow a bimodal distribution^9–11^. Consistent with this pattern, we found that the expression profiles from the three FFPE capture-based methods showed two major density peaks, with the first density peak of genes at 0 FPKM and the second at 1000 FPKM. Similarly, the distribution of gene expression of FF-TruSeq was bimodal, showing one peak density at 0 FPKM and the second peak at 100 FPKM (Supplementary Fig. 1c). Overall, the percentage of genes with no detectable expression was not significantly different between the three capture methods and the FF-TruSeq (Supplementary Fig. 1d). The FFPE capture-based methods captured a total of 17,801 genes that were common across all three methods. The unique genes that were captured by each method were 1880 for the Agilent platform, 360 for the TWIST platform, and 216 for the IDT platform (Supplementary Fig. 1e).

**Fig. 3:**
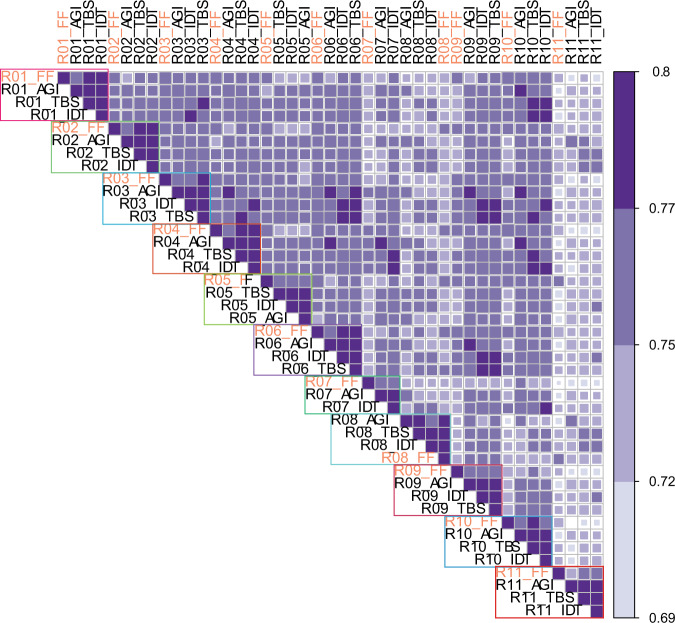
Heatmap showing the Spearman’s correlation coefficient of global mRNA expression. Each outlined block represents samples from the same patient. Color scale: 0.69 (lighter blue color) to 0.8 (darker blue color).

We then asked whether the expression profiles of the FFPE capture-based methods matched the FF-TruSeq profiles derived from the same samples. All the global expression profiles of the FFPE showed significant correlation with the corresponding FF-TrueSeq from the same tumor sample (Spearman’s *r* 0.72–0.90, *p* < 0.05) (Fig. 3) (Supplementary Data 3). In one patient (R11), the Agilent capture method showed a lower Spearman’s correlation of 0.72 with the corresponding FF-TruSeq-FF sample (*p* < 2.2e−16). Overall, the global gene expression pattern of FFPE tumor samples clustered with the corresponding FF sample in 8/11 of the matched sample sets in the t-distributed stochastic neighbor embedding plot (Supplementary Fig. 2). In only one patient (R11), the three FFPE capture-based methods did not cluster together (Supplementary Fig. 2). In addition, the three FFPE capture-based exome methods showed a significantly high correlation with each other (Spearman’s *r* range: 0.86–0.95) (Supplementary Data 3). These data suggest that the capture-based methods provide gene expression profiles that are consistent with those obtained from FF-TruSeq.

**Fig. 4:**
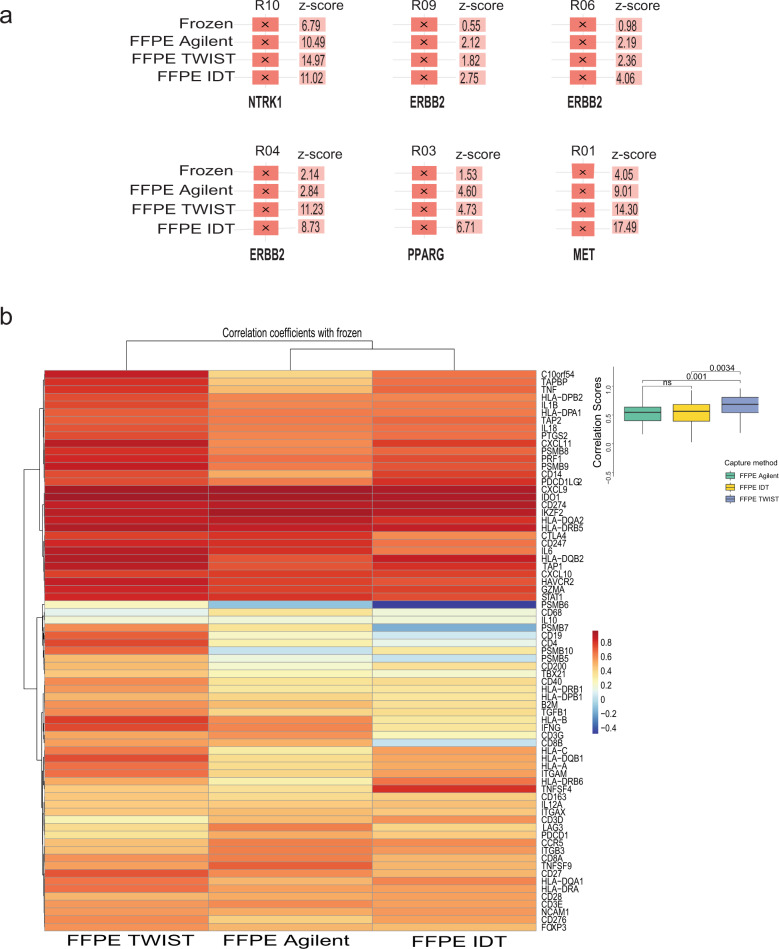
Gene expression outliers and immune gene correlation. **a** Outlier gene transcripts and z-scores in FF-TruSeq and the corresponding samples from the capture-based methods in the same patient. **b** Heatmap showing Spearman’s correlation coefficients based on gene expression of immune genes. Each cell represents the correlation coefficients of all the samples from a capture method with the corresponding FF-TruSeq. Overall, TWIST showed a higher correlation with FF-TruSeq.

Cancer cells exhibit outlier expression of several oncogenic transcripts. These overexpressed transcripts are potential therapeutic targets^12^. We examined the concordance of the expression of clinically relevant outlier genes in FF-TruSeq and whether the same outliers could be recovered from FFPE capture-based methods. For outlier detection, the mean and standard deviation of a gene were calculated across the Weill Cornell Medicine (WCM) RNA-seq cohort consisting of 650 multiple tumor samples. Outlier expression was defined as 1.5 times the interquartile range, z-score ≥ 2, and FPKM ≥ 20 (see “Methods” section). *ERBB2* was found to be an outlier in samples from three patients with urothelial cancer, including all three FFPE capture-based methods and FF-TruSeq (Fig. 4a). *MET, NTRK1*, and *PPARG* showed outlier expression in samples from three patients with GEJ adenocarcinoma, colorectal cancer, and urothelial cancer, respectively. We observed 100% concordance for outlier detection between FFPE capture-based methods and FF-TruSeq. These data suggest that FFPE capture-based methods provide a reliable tool for identifying clinically relevant expression outliers.

### Quantifying mRNA expression of immune-related genes

The characterization of immune cell infiltration using gene expression provides important information and has prognostic and predictive value in several cancer types^13^. For instance, the expression of immune-related genes correlates with response to immune checkpoint blockade in several cancers^13–16^. We quantified the concordance of the FPKM values of 73 key immune-related genes (see “Methods” section) between the FFPE exome capture-based and FF-TruSeq methods. A heatmap of the Spearman’s correlation scores across the expression profiles obtained from the three FFPE capture-based and FF-TruSeq methods is shown in Fig. 4b. Overall, the expression of individual gene transcripts from the FFPE capture-based methods correlated with the expression from the matching FF-TruSeq method. The expression of *PD-L1* (*CD274*) and *CTLA4* from the FF-TruSeq method significantly correlated with their expression from the Agilent method (*r* = 0.85, *p* = 0.002 and *r* = 0.83, *p* = 0.003), the IDT method (*r* = 0.87, *p* = 0.0009 and *r* = 0.88, *p* = 0.0006), and the TWIST method (*r* = 0.76, *p* = 0.01 and *r* = 0.88, *p* = 0.002), respectively (Supplementary Fig. 3). Overall, the TWIST method showed the highest correlation scores with the FF-TruSeq method, which were significantly higher than the Agilent (*p* = 0.001) and the IDT (*p* = 0.003) methods (Fig. 4b). These results suggest that FFPE exome capture-based methods provide a practical alternative to determine the expression of immune genes from tumor samples.

### mRNA expression-based molecular classification of urothelial cancers

A consensus mRNA expression-based single-sample classifier of muscle-invasive bladder cancers was recently published^17^. Applying this classifier to 18 datasets, six molecular classes were previously identified: luminal papillary (LumP), luminal nonspecified (LumNS), luminal unstable (LumU), stroma-rich, basal/squamous (Ba/Sq), and neuroendocrine-like (NE-like)^17^. To assess the applicability of using RNA-seq data from FFPE tumor samples for molecular classification consensus, we measured the concordance of the classifier outputs between the three FFPE capture-based and FF-TruSeq methods in five patients with urothelial cancer.

The three FFPE capture methods showed significant agreement with the FF-TruSeq method (50–80%) in classifying the molecular subtypes (Supplementary Data 4). The Cohen’s kappa for the agreement between the molecular class assignments was moderate to perfect for LumP (0.6), LumU (0.7), and Ba/Sq subtypes (1.00), but it was slight to poor for the stroma-rich (0.2) and LumNS (−0.1) subtypes. The NE-like subtype was not represented in our dataset.

### Fusion detection

**Fig. 5:**
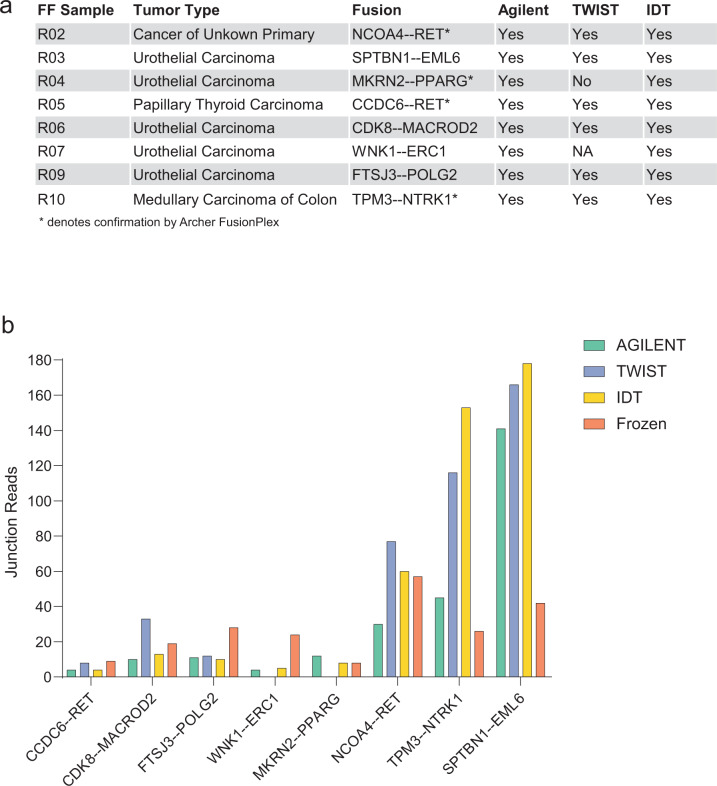
Detection of oncogenic gene fusions. **a** Table showing eight gene fusions that were identified in the FF tumor samples and their detection status with the corresponding capture-based methods. No samples were available for TWIST from one patient (R07). **b** Bar plots representing the number of the junction reads supporting the eight identified fusions derived from the FFPE capture-based and matching FF-TruSeq methods.

We evaluated the performance of the FFPE exome capture-based methods in detecting gene fusions compared to the FF-TruSeq method. In our cohort, we selected eight fusion transcripts that were initially identified in the FF tumor samples (see “Methods” section). Four fusions (*NCOA4-RET*, *CCDC6-RET*, *TPM3-NTRK1*, and *MKRN2-PPARG*) were orthogonally confirmed by targeted sequencing using the Archer FusionPlex platform from the FF samples^18^. The three FFPE capture-based methods successfully detected all the fusions except the *MKRN2-PPARG* fusion, which was missed by the TWIST capture method in one sample (Fig. 5a). In the FFPE tumor samples, the junction read count significantly correlated with the expression of the fusion transcripts (*r* = 0.95, *p* < 000.1). The Spearman’s correlation coefficients between junction read count and expression levels were 0.99 (Agilent, *p* < 0.0001), 0.92 (TWIST, *p* = 0.001), and 0.85 (IDT, *p* = 0.0034), respectively. The junction and spanning read counts supporting each fusion across the three capture methods were comparable with FF-TruSeq (Fig. 5b and Supplementary Data 5). Collectively, these data indicate that the FFPE capture-based methods can reliably identify the majority of fusions.

## Discussion

RNA-seq can simultaneously measure the expression of thousands of genes, provide composite readouts of critical signaling pathways, and detect oncogenic gene fusions. For these reasons, it provides a critical component of the precision medicine toolkit.

In this study, we performed transcriptomic profiling of FFPE tumor samples using three capture-based methods (Agilent, TWIST, and IDT). We benchmarked these methods to TruSeq from matching FF tumor samples. We tested these methods on a diverse tumor cohort chosen to represent tumors from real-world FFPE biobanks. This supports the generalizability of our results to different cancer types. Overall, the FFPE capture-based methods showed consistent performance in identifying biological signals, including outlier gene expression, oncogenic fusions, or quantifying the expression of key immune genes. On the other hand, more complex readouts, such as the molecular subtype classification were less consistent and thus need to be interpreted cautiously when using these platforms. The three capture-based methods successfully generated sequencing libraries from all tumor samples. The peak density of the DV200 and RIN were within the accepted quality range to proceed with library prep. Moreover, samples with low-quality metrics from degraded specimens did not adversely impact the sequencing output or the number of uniquely mapped reads using these methods. We observed that low DV200 and RIN values did not predict whether samples should be excluded from sequencing using these capture methods.

The minor differences in the total number of captured genes among the three capture-based methods did not significantly affect the global gene expression profiles. In fact, the global expression profiles of the FFPE capture-based methods positively correlated with the FF-TruSeq method across 11 matched tumor sets. We performed several downstream analyses to demonstrate the clinical utility of FFPE capture-based RNA-seq. We focused our analyses on clinically meaningful biological readouts, including the detection of expression outliers and oncogenic gene fusions, which are both amenable to therapeutic intervention. We also analyzed expression-based molecular subtyping of tumors which carries potential prognostic value^19^.

Identifying targetable outlier genes from RNA-seq has important clinical applications. *ERBB2* was identified as an outlier gene in three tumor sample sets from three urothelial cancer patients in our cohort. One patient showed an exceptional clinical response to trastuzumab following the detection of outlier *ERBB2* expression^20^. Outlier expression of the targetable oncogene *MET* was detected in a patient with GEJ adenocarcinoma and of *NTRK1* in a patient with medullary colon cancer, respectively. Capmatinib is a MET inhibitor -approved for non-small cell lung cancer patients^21^, and larotrectinib is an NTRK inhibitor for *NTRK* fusion-positive solid tumor patients^22^. Our data suggest that outlier gene expression measurements from FFPE samples can be potentially useful for identifying patients who may benefit from oncogene-targeted therapies.

Gene fusions are important therapeutic targets^23^. The detection of fusions from FFPE is potentially challenging because of low coverage and the potential for false-positive calls^2,4,24,25^. Interestingly, the three capture methods we tested identified all the clinically relevant fusions detected by FF-TruSeq except one fusion that was not captured by the TWIST platform. *RET* fusions were potentially clinically actionable in two patients with CUP and papillary thyroid cancer. In addition, an *NTRK1* fusion was identified in a colon cancer patient. *NTRK1* fusions are a tumor-agnostic marker with an FDA-approved indication for treatment with larotrectinib.

Our study opens the door to the interrogation of FFPE tissues from archival pathology repositories. Fixation and paraffin embedding are commonly used to preserve tissue morphology and enable histomorphological, immunohistochemical, and other in situ studies. A particular advantage of FFPE tissues is that they can be stored for a longer duration allowing the analysis of long-term patient outcomes^1^. The availability of FFPE-derived robust transcriptomic data will enable translational studies linking transcriptomic data to clinical phenotypes. This approach can also expand correlative studies to include FFPE tumor samples obtained from large multi-center clinical trials, mainly because many participating sites may not have the infrastructure for banking frozen tissues.

To the best of our knowledge, our study is the first to provide a comparison of three different FFPE capture-based methods applied to RNA from the same tumor sample. Previous reports attempted to examine the direct comparison of FF and individual FFPE capture methods from the same sample. The majority of these studies had smaller sample sizes (4–9 tumor samples)^4,26–28^ and were mainly focused on gene expression^26,28^.

Digital counting technologies (e.g., NanoString) can be used to interrogate FFPE samples. Unlike RNA-seq, which captures the expression of thousands of genes, these methods are currently restricted to a lower number of mRNA targets^29^. Using a dataset of 39 FFPE melanoma tumor samples, Kwong et al. compared RNA-seq to two NanoString gene expression panels^3^. They found that genes with low absolute expression showed poor correlation across platforms. This is consistent with our results across the FFPE capture-based methods suggesting that expression values of low abundance genes should be interpreted cautiously. We identified significant differences in molecular subtype membership assignment of urothelial cancers across the three capture-based methods, but this analysis was limited by the small number of urothelial cancers in the overall cohort. The current study reflects the tissue processing conditions at a single institution. The fixation and storage protocols in our study and the degree of degradation of FFPE samples may differ from those used by pathology departments at different institutions. The effects of these variations need to be studied. Another limitation of our study is that we did not evaluate all the available RNA exome platforms, such as the Illumina Exome library platform. Validation of our findings in multi-center studies that include diverse banking methods and different tissue types is warranted.

In conclusion, we compared three capture methods for transcriptomic profiling of FFPE tumors using a range of sequencing metrics and functional readouts. For outlier and immune gene expression, all capture-based methods demonstrated comparable performance. In other areas, namely, multigene-based subtyping and fusion detection, we identified platform-specific differences. Careful consideration of the biological and clinical questions and the intended use case would enable the optimal selection of the best-suited FFPE RNA capture method. Our results demonstrate the feasibility of using RNA exome capture-based methods and their broad clinical applications.

## Methods

### Sample collection

Patients signed informed consent (Weill Cornell Medicine IRB #1305013903). Banked excess tissue was collected from surgical specimens of patients with a diagnosis of cancer. All pathology specimens were reviewed by study pathologists (K.O., J.M.M.). Clinical charts were reviewed by the authors (K.S.S, J.M.M, B.M.F.) to record patient demographics, treatment history, anatomical site, and stage using the tumor, node, metastasis system published in the AJCC Cancer Staging Manual (8th edition).

### RNA extraction methods

For RNA extraction from FFPE tissues, the Maxwell 16 ® instrument with the Maxwell^®^ 16 LEV RNA FFPE Purification Kit was used as previously described^30^. This kit provides a high yield of pure RNA from FFPE tissue (and FF tissue, see below) samples. This protocol takes 60 min after macrodissection of the unstained FFPE slides and Proteinase K digestion to complete. Prior to macrodissection, hematoxylin and eosin (H&E) stained slides were cut and annotated by a pathologist to identify the tumor’s location in the corresponding unstained slides to be used in the extraction. Ten unstained slides of 10 µm thickness per case were cut for the extraction along with one H&E stained slide. The annotated locations on each slide were then macro-dissected with a sterile razor blade to obtain tissue for RNA extraction.

A side-by-side comparison using three specimens (R04, R08, and R11) was performed between the Promega Maxwell kit (https://www.promega.com/products/rna-purification-and-analysis/rna-purification/maxwell-16-lev-rna-ffpe-purification-kit/?catNum=AS1260), the Roche High Pure FFPET RNA isolation kit (https://lifescience.roche.com/en_us/products/high-pure-ffpet-rna-isolation-kit.html) and the Qiagen RNeasy FFPE kit (https://www.qiagen.com/us/shop/sample-technologies/rna/total-rna/rneasy-ffpe-kit/#orderinginformation). The RNA yields (ngs) and RIN numbers (~2.4) obtained from all three kits were similar. However, DV200 values were more variable. Two-thirds of the samples extracted using the Qiagen method had a DV200 <30. All the samples extracted using the Roche and Maxwell methods had DV200 >45. The Maxwell platform was chosen for extraction based on the availability of an automated workflow at our institution with the potential for scaling up the extraction of RNA from a large number of clinical samples. For extraction from frozen tissue, the Maxwell 16^®^ instrument with the Maxwell^®^ 16 LEV simplyRNA Tissue Kit was also used. Similarly, H&E stained slides were cut from the corresponding frozen block and annotated by a pathologist to identify the tumor location. Tissue from these annotated locations was removed using 1.5 mm diameter punch biopsies to core the block. Tissue homogenization was aided by introducing stainless steel beads to the tissue/homogenization solution and using the Qiagen Tissue Lyser LT set at 1/50 s for 2 min to physically break up the tissue before the lysis buffer was added.

### RNA quantity and quality assessment

The quantity of RNA was determined using a Nanodrop 2000 for nucleic acid absorbance measurement and a Qubit Fluorometer to confirm RNA concentration (ThermoFisher, Waltham, MA). Quality was assessed using a Bioanalyzer2100 (Agilent Technologies, Santa Clara, CA) with a high-sensitivity RNA chip. The RIN number was used to decide which RNA library prep kit to use for the frozen tissues; the DV_200_ measurement (the % of RNA fragments >200 nt) was used to determine the degree of RNA fragmentation for the FFPE samples (Evaluating RNA Quality from FFPE Samples. Illumina, Technical Note, publication number 470-2014 001. https://www.illumina.com/content/dam/illumina-marketing/documents/products/technotes/evaluating-rna-quality-from-ffpe-samples-technical-note-470-2014-001.pdf); SureSelectXT RNA Direct Protocol Provides Simultaneous Transcriptome Enrichment and Ribosomal Depletion of FFPE RNA, Agilent Technologies, Technical Note, publication number PR7000-0679. (https://www.agilent.com/cs/library/applications/5991-8119EN.pdf)

### To define the impact of the quality of the extracted RNA from FFPE samples on uniquely mapped reads

The relationship between two critical quality metrics was analyzed, the percentage of fragments >200 nucleotides (DV200 values) and RNA Integrity Number (RIN). For all FFPE tumor samples, DV200 and RIN ranged between 22 and 87 (median 45) and 2–2.7 (median 2.4), respectively (Supplementary Data 6 and 7, and Supplementary Fig. 4a, b). Across the same patient’s tumor samples, the RIN values significantly correlated with DV200 (Spearman’s *r* = 0.54, *p* = 0.001) (Supplementary Fig. 4c). Samples with DV200 20-30, or >30, had a similar degree of correlation with the number of uniquely mapped reads and the percentage of mapped reads. Overall, the DV200 showed no significant correlation with the number of mapped reads or the percentage of uniquely mapped reads (Spearman’s *r* = 0.13, and 0.16, *p* = 0.51 and 0.37, respectively) (Supplementary Fig. 5a, b), suggesting that low DV200 does not significantly impact the sequencing metrics of the FFPE capture-based methods. Similarly, the RIN value of each FFPE tumor sample did not lead to a significant difference among the uniquely mapped reads or the mapped reads percentage from the three FFPE capture-based methods (Spearman’s *r* = 0.12, and 0.12, *p* = 0.45 and 0.22, respectively) (Supplementary Fig. 5c, d). The initial RNA yields showed no significant correlation with the percentage of uniquely mapped reads (Spearman’s *r* = 0.13, *p* = 0.45). Both RIN and DV200 had a limited utility for excluding low-quality samples for exome capture-based methods.

The median FFPE block age was 3.25 years (range 1.6–4.9 years) (Supplementary Data 1). The FFPE blocks’ age was inversely correlated with DV200 (Spearman’s *r* = −0.45, *p* = 0.02). However, no significant correlation between blocks’ age and RIN (Spearman’s *r* = −0.01, *p* = 0.91) was observed. There was no significant correlation between FFPE blocks’ age with the percentage of uniquely mapped reads (Spearman’s *r* = −0.17, *p* = 0.39).

### RNA library preparation from fresh frozen tumor tissues

For RNA with RIN ≥ 6.0, libraries were prepared using TruSeq RNA Library Prep kit v2 (Illumina, San Diego, CA, PN-RS-122-2001). Briefly, poly A+ RNA was purified from 100 ng of total RNA with oligo-dT beads and fragmented to ~200 bp. cDNA was synthesized using random priming, then end-repair, dA-tailed, and ligated to Illumina TruSeq adaptors containing unique sequencing indexes. Libraries were amplified with 15 cycles of PCR and pooled for sequencing (Supplementary Data 6 and 7).

For RNA with RIN < 6, libraries were prepared with TruSeq Stranded Total RNA kit (Illumina, San Diego, CA, PN-20020596). Briefly, rRNA was depleted from 200 ng of total RNA with Ribo-Zero and fragmented to ~200 bp. cDNA was synthesized using random priming, and transcript orientation was preserved by using dUTP during second-strand cDNA synthesis. After end repair, A-tail, and ligation to Truseq adapters, libraries were generated by amplification with 15 cycles of PCR. Library pools were clustered at 6.5pM on a paired-end read flow cell and sequenced for 75 cycles on an Illumina HiSeq 2500 to obtain ~50 M reads per sample. (Supplementary Data 6 and 7).

### RNA-exome capture libraries

Briefly, stranded RNA-seq libraries were generated per the manufacturer’s recommendations but without the transcriptome enrichment step (pre-capture libraries). Transcriptome enrichment was achieved by the hybridization of the pre-capture library to the exome panels tested. Since the probe baits were biotinylated, hybridized libraries were captured using streptavidin beads (ThermoFisher, Waltham, MA) and PCR amplified-on-beads to generate a post-capture library. All post-capture libraries were subjected to quality control on an Agilent Bioanalyzer and normalized to 2 nM. The post-capture libraries obtained from each capture platform were pooled, and each pool was sequenced on one lane of a paired-end read flow cell for 2 × 100 cycles on a HiSeq4000 to obtain ~40 M reads per sample. The primary processing of sequencing images was done using Illumina’s Real Time Analysis software. CASAVA 1.8.2 software was then used to demultiplex samples and generate raw reads and respective quality scores (Supplementary Data 6 and 7). For samples with DV200 <30, additional PCR cycles above the number recommended in the manufacturers’ technical notes were added (Supplementary Data 7).

Sure Select^XT^ Human All exon v6+UTRs (PN-5190-881, Agilent, Santa Clara, CA): Non-indexed pre-capture libraries were made using SureSelect ^XT^ RNA Direct kit (PN-G9691-90050) with 200 ng of RNA, using the % DV_200_ obtained with the Agilent Bioanalyzer to determine fragmentation times and amplifying 14–16 PCR cycles. Hybridization was carried out with 200 ng from each pre-capture library for 24 h × 65 °C on RNA-biotinylated probes. Indexes were added during post-capture libraries amplification using 12 cycles.

Twist Human Core Exome (PN100790, Twist Biosciences, San Francisco, CA): Libraries were made using the NEBNext Ultra II Directional kit (PN-E7760, New England Biolabs, Ipswich, MA) with 100 or 200 ng depending on the % DV_200_ of the starting material. Pre-capture libraries were uniquely indexed for Illumina sequencing, using 11–16 amplification cycles. A total of 1.5 µg of pooled indexed libraries (300 ng each, two pools) were hybridized to the biotinylated double-stranded DNA probe panel for 16 h at 70 °C. Post-capture libraries were amplified for eight cycles (DOC-001014).

IDT xGen Exome Research Panel v1.0 (Integrated DNA Technologies, Coralville, IA): Libraries were made as described above using the NEBNext Ultra II Directional kit (PN-E7760, New England Biolabs, Ipswich, MA). A total of 5 µg of pooled indexed libraries (500 ng each) were hybridized to the biotinylated oligo probes for 4 h at 65 °C. Post-capture libraries were amplified for seven cycles (NGS-10122-PR 01/2020).

The Agilent capture-based method targets 91 Mb of genomic DNA sequence in addition to 5′ and 3′ UTR sequences. IDT and TWIST methods target 39 and 33 Mb of the coding sequences (CDS) of human coding genes, respectively. The three capture-based methods use 120-base RNA probes to capture known CDS. The total number of captured genes is 20,456 for Agilent, 19,075 for IDT, and 19,542 for TWIST.

### RNA sequencing analysis

All reads were independently aligned with STAR_2.4.0f1^31^ for sequence alignment against the human genome sequence build hg19, downloaded via the UCSC genome browser [http://hgdownload.soe.ucsc.edu/goldenPath/hg19/bigZips/], and SAMTOOLS v0.1.19^32^ for sorting and indexing reads. Cufflinks (2.0.2)^33^ was used to estimate the expression values (FPKMS) and GENCODE v19^34^ GTF file for annotation. Since the sequenced samples were processed using different library preps, batch normalization of FPKMs from WCM Frozen samples was done using ComBat from the sva Bioconductor package^35^. For fusion analysis, we used STAR-fusion (STAR-Fusion_v0.5.1)^36,37^. Fusions with significant support of junction reads (≥1) and spanning pairs (≥1) were selected. For outlier detection, the FPKMs from batch normalized frozen WCM samples were combined with the FPKMs from FFPE samples. We only selected the druggable genes from drugbank^38^ as well as cancer genes from Oncokb^39^, which resulted in a list of 138 druggable cancer genes. The mean and standard deviation of each gene were calculated across the WCM RNA-seq cohort (multiple cancer types). An outlier was defined as having 1.5 times the interquartile range, z-score ≥2, and FPKMs ≥20.

### Statistical analysis

For pairwise comparisons, we used the Wilcoxon signed-rank test. For comparison of the post-alignment statistics among the three capture methods, the Kruskal–Wallis test was performed. Correlation analyses between gene expression profiles were performed using the Spearman’s rank correlation test. To measure the inter-classifier concordance, the Cohen’s kappa statistic measure of inter-rater agreement was calculated. The kappa-statistic measure of agreement was scaled to 0 when the amount of agreement is what would be expected to be observed by chance and 1 when there is perfect agreement. We used the Landis and Koch method^40^, which suggests the following interpretations. Below 0.0: Poor, 0.00–0.20: Slight, 0.21–0.40: Fair, 0.41–0.60: Moderate, 0.61–0.80: Substantial, 0.81–1.00: Almost perfect. RStudio (1.0.136) with R (v3.3.2) and ggplot2 (2.2.1) were used for statistical analysis and generating plots. A *p* value <0.05 was considered significant. All tests were two-sided.

### Reporting summary

Further information on research design is available in the Nature Research Reporting Summary linked to this article.

## Supplementary information

Supplementary Data 1

Supplementary Data 2

Supplementary Data 3

Supplementary Data 4

Supplementary Data 5

Supplementary Data 6

Supplementary Data 7

Supplementary Information

Reporting Summary

## Data Availability

The raw RNA-seq datasets analyzed during the current study are available in the European Genome-phenome Archive (EGA). The FASTQ files and associated sample information are deposited in the European Genome-phenome Archive (EGA) under the accession number (EGAS00001005255).

## Code availability

All analyses in this study were performed using R version 3.3.2, ggplot version 2.2.1, SAMTOOLS v0.1.19, Cufflinks (2.0.2), GENCODE v19, or STAR-Fusion_v0.5.1., and the sva Bioconductor package. Custom R scripts used for the individual analyses are available upon request.
