# Supplementary material for: Functional comparison of exome capture-based methods for transcriptomic profiling of formalin-fixed paraffin-embedded tumors
Source: NPJ Genom Med. 2021 Aug 12;6:66. doi: 10.1038/s41525-021-00231-7 (PMC8360986; doi:10.1038/s41525-021-00231-7)
Supplement: Supplementary file 8 — Supplementary Information [file 41525_2021_231_MOESM8_ESM.pdf]

Supplementary Information

Functional Comparison of Exome Capture-based Methods for Transcriptomic Profiling of Formalin-Fixed Paraffin-Embedded Tumors

Shohdy K et al.

Supplementary Figures

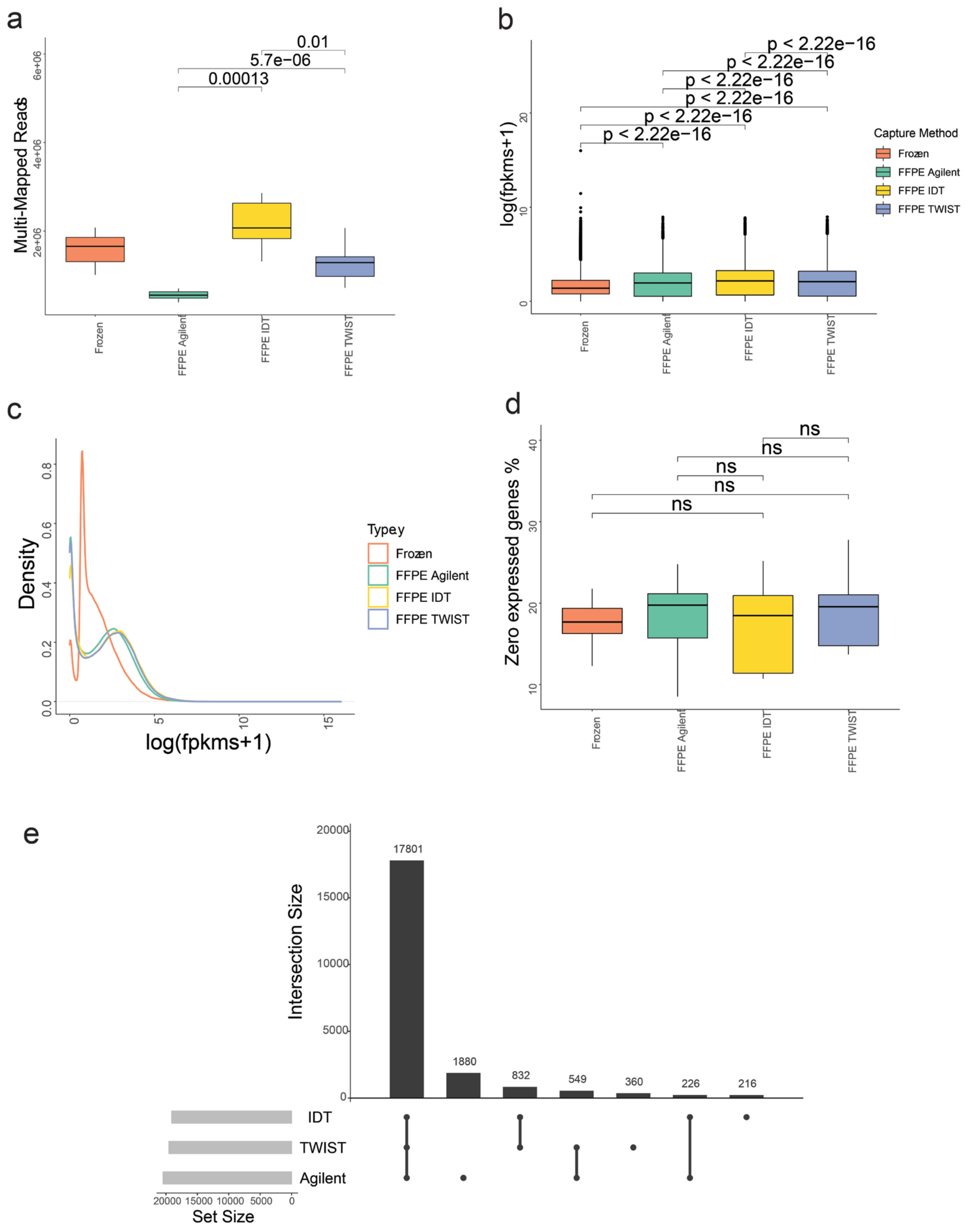

Supplementary Figure 1. (a) Boxplots of multi-mapping reads comparing the FF-TruSeq and the exome capture methods (Wilcoxon test). (b) Boxplot of log (FPKMS + 1) comparing frozen and the capture methods (Wilcoxon test). (c) Density plot of log (FPKMS +1) comparing frozen and the capture methods. (d) Boxplots of the number of zero expressed genes comparing the frozen and the exome capture methods (Wilcoxon test). (e) Upset plot of the number of genes in the bed files of the capture-based methods showing the overlapping number of captured genes.

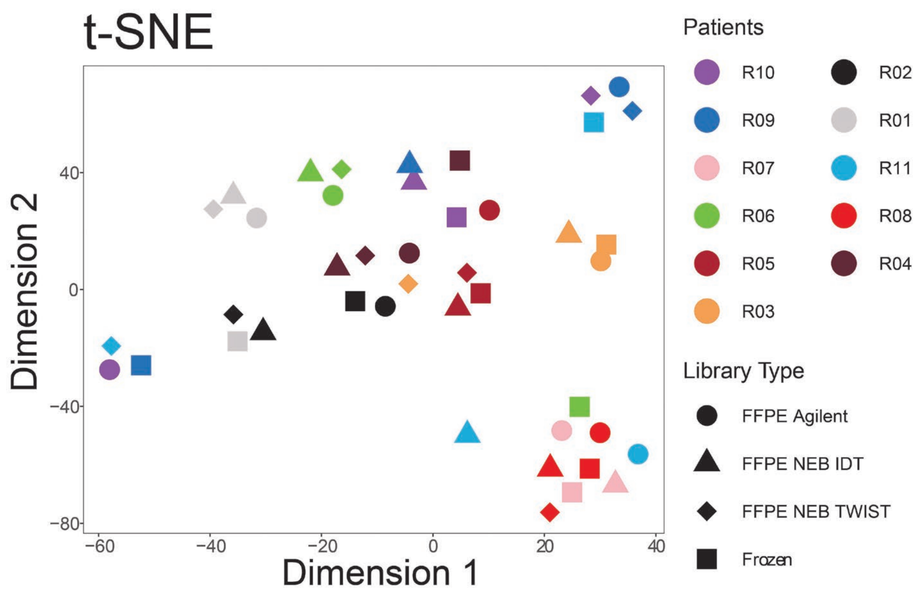

Supplementary Figure 2. t-SNE projection of samples based on whole transcriptomic profiles (all coding protein-coding genes) colored by patients (shapes represent different capture methods) showing clustering of FFPE capture-based methods with the FF-TruSeq matched samples.

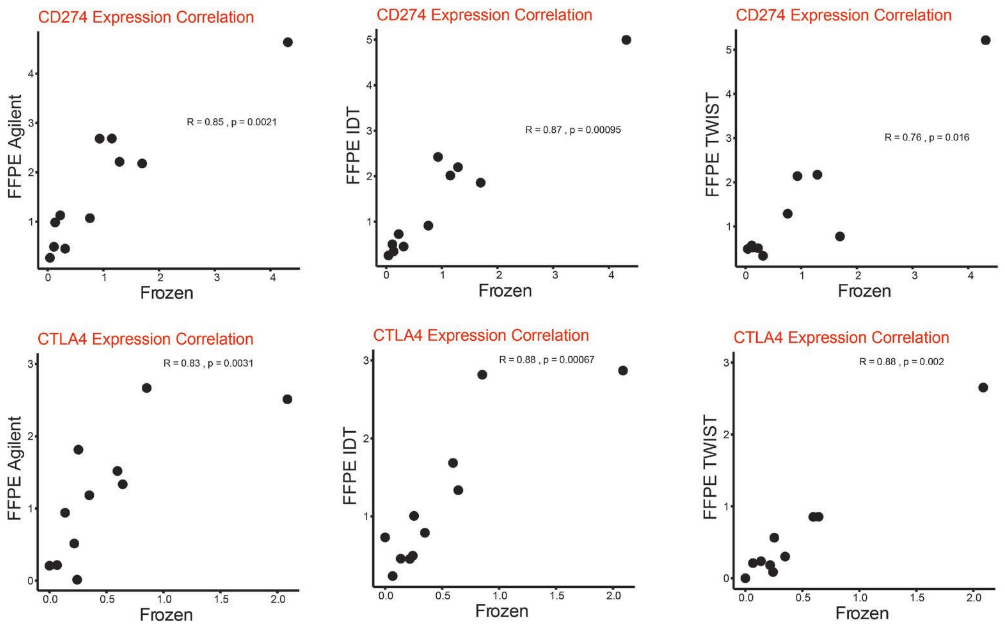

Supplementary Figure 3. A significant correlation of the FPKM expression of *CD274* (PD-L1) and *CTLA4* was observed between FF-TruSeq, and the matching three capture-based methods.

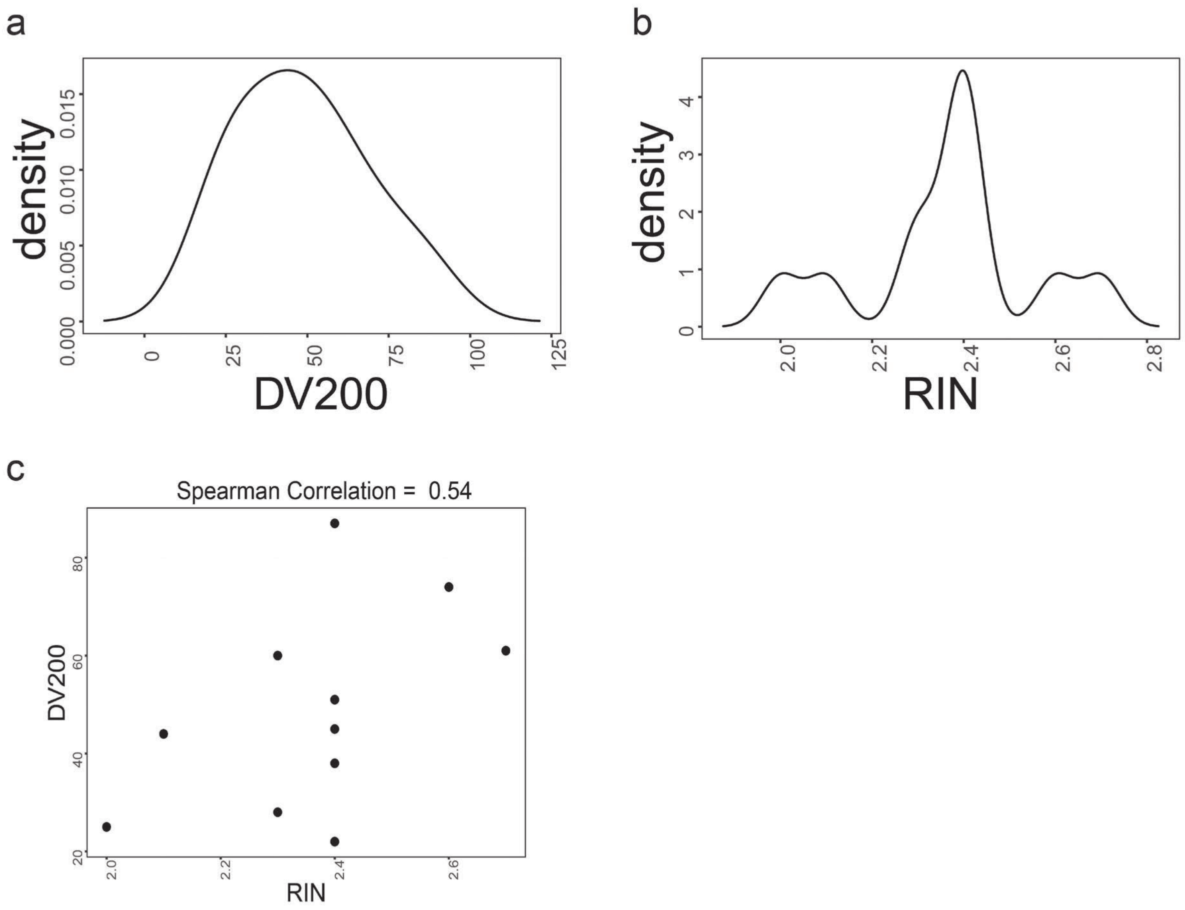

Supplementary Figure 4. **Quality Control Metrics.** (a) Density plot of DV200. (b) Density plot of RNA integrity number (RIN). (c) Correlation between DV200 and RIN (Spearman's correlation = 0.54).

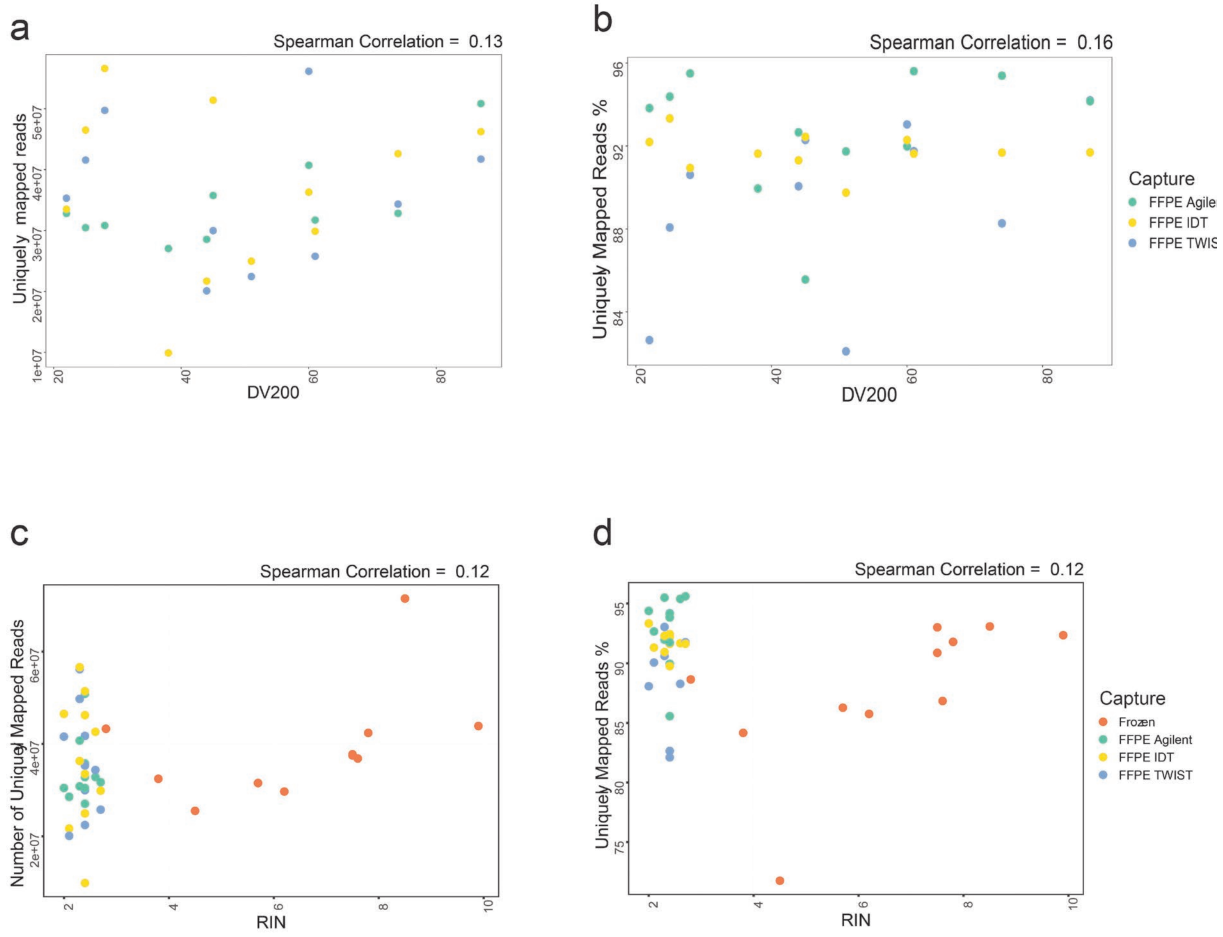

Supplementary Figure 5. Alignment statistics and correlation with RNA quality control metrics. (a) Correlation between DV200 and uniquely mapped reads (Spearman's Correlation = 0.13, p = 0.51). (b) Correlation between DV200 and Uniquely Mapped Reads % (Spearman's Correlation = 0.16, p = 0.37). (c) Correlation between RIN and Uniquely Mapped Reads (Spearman's Correlation = 0.12, p = 0.45). (d) Correlation between RIN and uniquely mapped reads percentages (Spearman's Correlation = 0.12, p = 0.22).

Additional Files:

Supplementary Data 1: Clinical and pathologic characteristics of patients and their individual samples.

Supplementary Data 2: Alignment statistics of FFPE capture-based methods and the matched FF-TruSeq samples.

Supplementary Data 3: The Spearman's r squared for the correlation among the global expression profiles of the three FFPE capture-based exome methods and the FF-TruSeq.

Supplementary Data 4: Agreement between the consensus molecular classifier assignments using RNA-seq data from FF-TruSeq and matched FFPE capture-based methods from the bladder cancer samples.

Supplementary Data 5: Junction read counts of the identified gene fusions in FF-TruSeq tumor samples and matched FFPE exome capture-based methods.

Supplementary Data 6: Workflow of RNA sequencing for fresh frozen and FFPE tumor samples.

Supplementary Data 7: Quality metrics of RNA library preparation from fresh-frozen and FFPE samples.
