# Supplementary material for: Functional comparison of exome capture-based methods for transcriptomic profiling of formalin-fixed paraffin-embedded tumors
Source: NPJ Genom Med. 2021 Aug 12;6:66. doi: 10.1038/s41525-021-00231-7 (PMC8360986; doi:10.1038/s41525-021-00231-7)
Supplement: Supplementary file 9 — Reporting Summary [file 41525_2021_231_MOESM9_ESM.pdf]

nature research

Corresponding author(s): Bishoy Faltas

Last updated by author(s): Apr 20, 2021

Reporting Summary

Nature Research wishes to improve the reproducibility of the work that we publish. This form provides structure for consistency and transparency in reporting. For further information on Nature Research policies, see our Editorial Policies and the Editorial Policy Checklist.

Statistics

For all statistical analyses, confirm that the following items are present in the figure legend, table legend, main text, or Methods section.

n/a	Confirmed	
☐	☒	The exact sample size (n) for each experimental group/condition, given as a discrete number and unit of measurement
☐	☒	A statement on whether measurements were taken from distinct samples or whether the same sample was measured repeatedly
☐	☒	The statistical test(s) used AND whether they are one- or two-sided<br>*Only common tests should be described solely by name; describe more complex techniques in the Methods section.*
☐	☒	A description of all covariates tested
☐	☒	A description of any assumptions or corrections, such as tests of normality and adjustment for multiple comparisons
☐	☒	A full description of the statistical parameters including central tendency (e.g. means) or other basic estimates (e.g. regression coefficient) AND variation (e.g. standard deviation) or associated estimates of uncertainty (e.g. confidence intervals)
☐	☒	For null hypothesis testing, the test statistic (e.g. F, t, r) with confidence intervals, effect sizes, degrees of freedom and P value noted<br>*Give P values as exact values whenever suitable.*
☒	☐	For Bayesian analysis, information on the choice of priors and Markov chain Monte Carlo settings
☒	☐	For hierarchical and complex designs, identification of the appropriate level for tests and full reporting of outcomes
☐	☒	Estimates of effect sizes (e.g. Cohen's d, Pearson's r), indicating how they were calculated

Our web collection on statistics for biologists contains articles on many of the points above.

Software and code

Policy information about availability of computer code

Data collection	no software was used
Data analysis	All reads were independently aligned with STAR_2.4.0f1 for sequence alignment against the human genome sequence build hg19, downloaded via the UCSC genome browser [http://hgdownload.soe.ucsc.edu/goldenPath/hg19/bigZips/], and SAMTOOLS v0.1.19 for sorting and indexing reads. Cufflinks (2.0.2) was used to estimate the expression values (FPKMS), and GENCODE v1913 GTF file for annotation. Since the sequenced samples were processed using different library preps, batch normalization of FPKMs from WCM Frozen samples was done using ComBat from the sva Bioconductor package. For fusion analysis, we used STAR-fusion (STAR-Fusion_v0.5.1). Fusions with significant support of junction reads (≥1) and spanning pairs (≥1) were selected. For outlier detection, the FPKMs from batch normalized frozen WCM samples were combined with the FPKMs from FFPE samples. We only selected the druggable genes from drugbank as well as cancer genes from Oncokb, which resulted in a list of 138 druggable cancer genes. The mean and standard deviation of each gene were calculated across the WCM RNA-seq cohort (multiple cancer types). An outlier was defined as having 1.5 times the interquartile range, z-score ≥2, and FPKMs ≥20.

For manuscripts utilizing custom algorithms or software that are central to the research but not yet described in published literature, software must be made available to editors and reviewers. We strongly encourage code deposition in a community repository (e.g. GitHub). See the Nature Research guidelines for submitting code & software for further information.

Data

Policy information about availability of data

All manuscripts must include a data availability statement. This statement should provide the following information, where applicable:

- Accession codes, unique identifiers, or web links for publicly available datasets
- A list of figures that have associated raw data
- A description of any restrictions on data availability

The raw RNA-seq datasets analyzed during the current study are available in the European Genome-phenome Archive (EGA). The FASTQ files and associated sample information are deposited in EGA under the accession number (EGAS00001005255).

Field-specific reporting

Please select the one below that is the best fit for your research. If you are not sure, read the appropriate sections before making your selection.

☒ Life sciences ☐ Behavioural & social sciences ☐ Ecological, evolutionary & environmental sciences

For a reference copy of the document with all sections, see nature.com/documents/nr-reporting-summary-flat.pdf

Life sciences study design

All studies must disclose on these points even when the disclosure is negative.

Sample size	The study included 11 patients with cancer from Weill Cornell Medicine (WCM). A cohort of 32 formalin-fixed, paraffin-embedded (FFPE) tumor samples from the 11 patients was collected. For each patient, a matched fresh frozen (FF) tumor sample was available.
Data exclusions	no data were excluded from analysis
Replication	The FFPE tumor samples were analyzed using three commercially available FFPE capture-based methods (Agilent, TWIST, IDT), to allow comparison of performance characteristics of the three methods
Randomization	No randomization was adopted.
Blinding	Blinding was not relevant to our analysis, the study investigated molecular characteristics and did not apply experimental interventions to the studied subjects.

Reporting for specific materials, systems and methods

We require information from authors about some types of materials, experimental systems and methods used in many studies. Here, indicate whether each material, system or method listed is relevant to your study. If you are not sure if a list item applies to your research, read the appropriate section before selecting a response.

Materials & experimental systems

n/a	Involved in the study	
☒	☐	Antibodies
☒	☐	Eukaryotic cell lines
☒	☐	Palaeontology and archaeology
☒	☐	Animals and other organisms
☐	☒	Human research participants
☒	☐	Clinical data
☒	☐	Dual use research of concern

Methods

n/a	Involved in the study	
☒	☐	ChIP-seq
☒	☐	Flow cytometry
☒	☐	MRI-based neuroimaging

Human research participants

Policy information about studies involving human research participants

Population characteristics	We included 11 patients with histologically-proven cancer. We included several tumor types, namely urothelial cancer, gastroesophageal junction (GEJ) adenocarcinoma, oligodendroglioma, cancer of unknown primary (CUP), leiomyosarcoma, papillary thyroid cancer, and colorectal cancer.
Recruitment	Patients signed informed consent (Weill Cornell Medicine IRB #1305013903). Banked excess tissue was collected from surgical specimens of patients with a diagnosis of cancer. All pathology specimens were reviewed by study pathologists (K.O., J.M.M). Clinical charts were reviewed by the authors (K.S.S, J.M, B.M.F.) to record patient demographics, treatment history,

	anatomical site, and stage using the tumor, node, metastasis (TNM) system published in the AJCC Cancer Staging Manual (8th edition).
Ethics oversight	Weill Cornell Medicine IRB

Note that full information on the approval of the study protocol must also be provided in the manuscript.
